# Reduced 2-year aneurysm retreatment and costs among patients treated with flow diversion versus non-flow diversion embolization: A Premier Healthcare Database retrospective cohort study

**DOI:** 10.1371/journal.pone.0234478

**Published:** 2020-06-18

**Authors:** Ramesh Grandhi, Michael Karsy, Philipp Taussky, Christine Nichols Ricker, Ajay Malhotra

**Affiliations:** 1 Department of Neurosurgery, Clinical Neurosciences Center, University of Utah, Salt Lake City, Utah, United States of America; 2 Medtronic Pain Therapies, Fridley, Minnesota, United States of America; 3 Department of Radiology and Biomedical Imaging, Yale School of Medicine, New Haven, Connecticut, United States of America; University of Florida, UNITED STATES

## Abstract

**Introduction:**

The use of endovascular treatments, including Pipeline embolization devices (PEDs) and coiling approaches (non-PEDs), has played an increasingly important role in the treatment of intracranial aneurysms. Despite multiple studies evaluating PEDs, a real-world evaluation of follow-up outcomes and costs remains to be completed.

**Methods:**

The Premier Healthcare Database (PHD), 2010–2017, was queried to identify patients with unruptured intracranial aneurysms treated endovascularly. Rates of readmission, retreatment, and cost at the same hospital were compared between patients who underwent PED and non-PED endovascular treatments of their aneurysms. One-to-three (PED–to–non-PED) propensity score (PS) matching was performed to adjust for potential case selection bias into the PED cohort, with covariates including age group, sex, Charlson Comorbidity Index (CCI) group, payor, region, and randomized hospital identifier.

**Results:**

A total of 679 patients underwent PED placement and 8432 had non-PED treatments. Prior to PS matching, there were significant but minor differences in age (56.7±12.8 vs. 58.2±12.6 years, p = 0.004) and sex (male 16.6% vs. 24.4%, p<0.0001) for PED and non-PED, respectively, but no differences in CCI (p = 0.08), length of stay (p = 0.88), or rate of routine discharge (p = 0.21). All-cause readmission/emergency department reevaluation rates in the two cohorts were similar at 30, 90, and 180 days and 1 and 2 years. Our results identified a significantly lower retreatment rate for PEDs at all follow-up time points over a 2-year period (range: 0.9–8.1%) compared with non-PED treatments (range: 1.7–11.6%). These findings remained consistent after PS matching: all-cause readmission/reevaluation rates were significantly lower in patients treated with PED at 90 days, 180 days, 1 year, and 2 years (p<0.001). Although the initial treatment costs were higher for PED at time of treatment (p<0.001), cumulative follow-up emergency department visit and readmission costs (inclusive of patients with no readmission and/or no retreatment) were significantly lower for patients with initial PED relative to non-PED treatment at 2 years (p = 0.021).

**Conclusions:**

These results suggest that PEDs may potentially reduce downstream retreatment rates and costs. Further work is required to improve identification of patient subgroups that could benefit from PED over non-PED treatments both initially and during follow-up.

## Introduction

Intracranial aneurysms represent a relatively common intracranial disease, with a prevalence of 3–11% and rupture rate of roughly 3–5% per year [1–3]. The advent of minimally invasive, catheter-based endovascular approaches for aneurysm treatment has represented a significant change in the way aneurysms are treated in the modern healthcare setting [4–7]. The results of the Analysis of Treatment by Endovascular approach of Non-ruptured Aneurysms (ATENA) [8], the Internal Subarachnoid Trial (ISAT) [9], the Clinical and Anatomical Results in the Treatment of Ruptured Intracranial Aneurysms (CLARITY) trial [10], and the Barrow Ruptured Aneurysm Trial (BRAT) [11], combined with advancements in neurointerventional technologies and techniques, including improvement in coil technology, development of balloons and stents for use as adjuncts during coil embolization procedures, endosaccular devices, and flow diverters, have influenced the management strategy of practitioners caring for patients with unruptured and ruptured intracranial aneurysms [6]. The introduction of the Pipeline^TM^ Embolization Device (PED) (Medtronic, Irvine, CA, USA), in particular, has been a disruptive innovation in the field of cerebrovascular surgery for the treatment of intracerebral aneurysms [12–15]. The PED was initially approved by the U.S. Food and Drug Administration in 2011 for the treatment of large, wide-necked aneurysms, with the indication more recently expanded in 2018 to include small internal carotid artery aneurysms. Off-label uses (e.g., for smaller aneurysms) are also widely reported and have shown clinical efficacy with good outcomes. Nevertheless, the treatment of intracranial aneurysms remains challenging, because of the possibility of intraprocedural complications and also delayed postprocedural complications with consequent need for readmission, in addition to the potential need for retreatment because of aneurysm residual and regrowth [16, 17].

The Premier Healthcare Database (PHD) is a U.S. hospital-based all-payor database with detailed information on inpatient admissions that has previously been used in one study of PED treatment among patients with intracranial aneurysms [18]. A significant limitation of the current literature is the lack of head-to-head comparison of the various neurointerventional techniques in a large-scale, multicenter, real-world setting including evaluation of patients over follow-up. Thus, important considerations such as rates of readmission after aneurysm treatment, rates of retreatment, and consequent costs have not been well elucidated. We aimed to evaluate rates of readmission and retreatment of unruptured aneurysms among patients in whom PED was used relative to patients treated with non-PED endovascular modalities using this large-scale, multicenter database. The secondary aim was to study hospital costs associated with each treatment strategy.

## Materials and methods

### Study patients

The PHD includes diagnosis, procedure, and product-level information pertaining to 108 million unique inpatient visits from 970 hospitals, which represents approximately 25% of annual admissions in the U.S. [19]. Hospitals included in PHD comprise teaching/academic facilities as well as community-based hospitals from geographically diverse regions, including both urban and rural environments. The PHD provides hospital-level encounter data tracking patients through inpatient, outpatient hospital, and emergency department visits.

No Internal Review Board approval was required for this study because the data used for analyses were deidentified. Data were abstracted from the PHD from 2010 through 2017. International Classifications of Diseases, Ninth Revision, Clinical Modification (ICD9-CM) code 437.3 (cerebral aneurysm, unruptured) and ICD10-CM code I67.1 (cerebral aneurysm, nonruptured) were used to identify patients with an admitting or principal diagnosis of unruptured intracranial aneurysm. Patients were included if they were ≥18 years of age and underwent an endovascular treatment for an intracranial aneurysm (defined as presence of ICD9-CM procedure codes 39.72, 39.75, 39.76 or ICD10-CM procedure codes 03VG3BZ or 03VG3DZ, or Current Procedural Terminology code 61624). Patients classified by ICD10-CM codes 747.81 (anomalies of cerebrovascular system) or Q28.3 (other malformations of cerebral vessels) were excluded because it was unclear whether a “true” intracranial aneurysm was present in the absence of further clinical detail from imaging. Finally, patients with diagnosis of intracerebral, intracranial, or subarachnoid hemorrhage during the hospitalization were excluded from analyses given the inherently different patient severity profile. Endovascular PED treatment and non-PED treatments (e.g., coiling alone, stent-assisted coiling, balloon-assisted coiling) were compared. PED was identified via product names listed in the Chargemaster file, whereas the non-PED cohort was defined as the remaining patients meeting study selection criteria who had no evidence of PED treatment recorded. We were unable to delineate the specific non-PED treatment (e.g., coiling-only, stent-assisted coiling, balloon-assisted coiling) due to limitations in the specificity of ICD9 and ICD10 procedure coding over the years of data evaluated, and limitations in sensitivity of product names for stent and balloon-related products listed in the Chargemaster file.

Among the patients meeting the selection criteria, one PED patient was matched to three non-PED patients using a propensity score (PS) model with the nearest neighbor approach and caliper width of ±0.10 of the standard deviation of the propensity score. Covariates in the propensity model included age group, sex, Charlson Comorbidity Index (CCI, a measure of patient comorbidity), region, primary payor, and the hospital randomized facility ID. Patients were matched by facility ID to ensure comparison of patients initially treated within the same hospital, but with different treatment approaches, to account for hospital-specific variations in practice patterns and costs.

### Study variables

Patient demographic variables included age, sex, CCI, and payor type. The length of stay and discharge disposition were summarized for the initial admission. Cumulative incidence of follow-up all-cause readmissions and readmission for embolization retreatment were evaluated at 30 days, 90 days, 180 days, 1 year, and 2 years, among patients returning to the same hospital. Given the primarily cross-sectional nature of the Premier Healthcare Database, only readmissions to the initial treatment hospital were captured.

The total hospital cost of the initial admission and the readmission cost specific to retreatment were summarized by study cohort. Additionally, total follow-up costs for all readmissions (regardless of retreatment) were averaged through one- and two-year follow-up across the entire study cohort, regardless of whether a readmission occurred (i.e., inclusive of patients with no readmission). Cost was defined as internal hospital costs (as opposed to charges or payment/reimbursement amounts).

### Statistical analysis

Continuous variables are reported as means ± standard deviation (STD) whereas discrete variables are reported as number and % of total. The comparison of statistical significance across cohorts was calculated with the t-test for normally distributed continuous variables, the Wilcoxon-Mann-Whitney test for skewed (cost) variables, and the chi-squared test for discrete variables. A p<0.05 was considered statistically significant. Sample selection and creation of analytic variables were performed using the Instant Health Data (IHD) platform (BHE, Boston, MA, USA). Statistical analyses were undertaken with R, version 3.2.1 (R Foundation for Statistical Computing, Vienna, Austria).

## Results

A total of 679 patients who underwent PED treatment and 8432 patients who underwent non-PED treatment (i.e., coiling, balloon-assisted coil embolization, or stent-assisted coil embolization) were identified (Table 1). These groups had significantly different baseline age (56.7±12.8 vs. 58.2±12.6 years, p = 0.004) and sex (male; 16.6% vs. 24.4%, p<0.0001) for PED and non-PED, respectively. No differences in CCI group (p = 0.08), length of stay (3.5±3.7 vs. 3.5±3.8 days, p = 0.9), or rate of routine discharge disposition (92.5% vs. 91.0%, p = 0.2) were observed for PED and non-PED, respectively.

**Table 1:** Baseline demographics and pre-matched analysis of patients with intracerebral aneurysms treated endovascularly.

Variable	PED N = 679	Non-PED N = 8432	P-value
Age (mean±STD; median), years	56.7±12.8; 57	58.2±12.6; 59	0.004
Sex (% total), male	113 (16.6)	2057 (24.4)	<0.0001
Payor (% total)			0.0001
Commercial	351 (51.7)	3466 (41.1)	
Medicaid	80 (11.8)	885 (10.5)	
Medicare	220 (32.4)	3415 (40.5)	
Other	28 (4.1)	666 (7.9)	
Charlson Comorbidity Score			0.08
0	457 (67.3)	5321 (63.1)	
1	129 (19.0)	1745 (20.7)	
2+	93 (13.7)	1374 (16.3)	
Length of stay (mean±STD; median), days	3.5±3.7; 2	3.5±3.8; 2	0.9
Routine disposition	628 (92.5)	7682 (91.1)	0.2
All-cause readmissions including embolization			
30 days	50 (7.4)	539 (6.6)	0.5
90 days	81 (11.9)	1095 (13.5)	0.3
180 days	113 (16.6)	1451 (17.9)	0.5
1 year	151 (22.2)	1874 (22.8)	0.8
2 years	170 (25.0)	2247 (27.4)	0.2
Readmission for repeat embolization			
30 days	6 (0.9)	133 (1.7)	0.1
90 days	18 (2.7)	456 (5.7)	0.001
180 days	18 (4.7)	448 (7.9)	0.004
1 year	48 (7.1)	852 (10.1)	0.01
2 years	55 (8.1)	978 (11.6)	0.007

**Fig 1:**
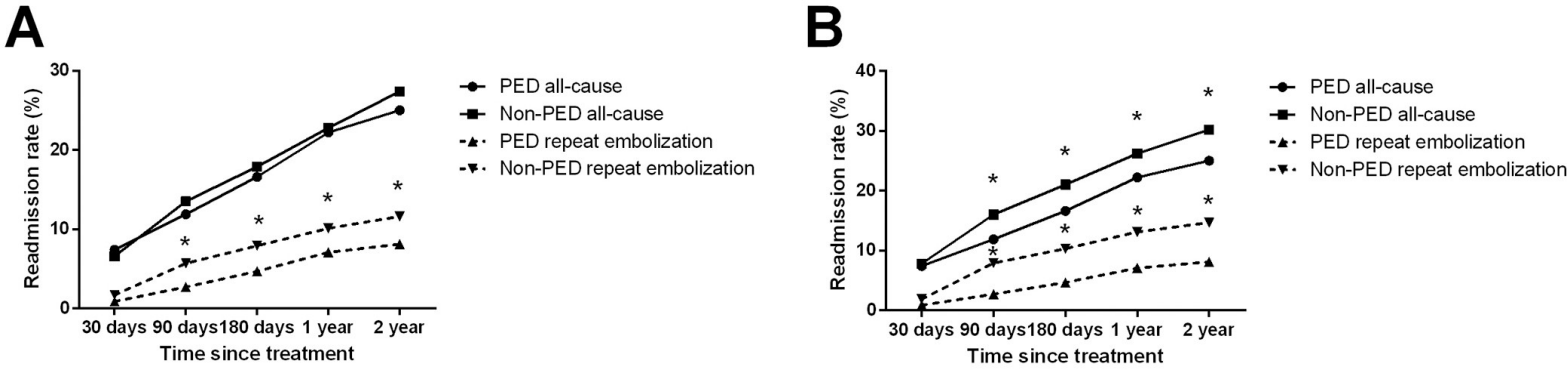
All-cause and repeat embolization outcomes after PED or non-PED treatment during two-year follow-up. (A) The percentage of all-cause and repeat embolization outcomes are shown up to two-year follow-up. A significant reduction in repeat embolization was seen in PED treatments. (B) All-cause and repeat embolization outcomes during two-year follow-up are seen after propensity match analysis. Here, a significant reduction in all-cause and repeat embolization outcomes are seen for PED treatment. P<0.05.

Prior to PS matching, no difference in all-cause readmission rate was seen at up to 2-year follow-up (Table 1, Fig 1A). Cumulative repeat embolization rates were significantly lower in the PED vs. non-PED cohorts at 90 days (2.7% vs. 5.7%, p = 0.001), 180 days (4.7% vs. 7.9%, p = 0.004), 1 year (7.1% vs. 10.1%, p = 0.01), and 2 years (8.1% vs. 11.6%, p = 0.007) follow-up. In other words, at 90-day, 180-day, 1-year, and 2-year follow-up, patients who underwent PED treatment had a 3.0%, 3.2%, 3.2%, and 3.5% lower rate of readmission for embolization, respectively.

After PS matching, the 679 PED treated patients were matched to 1918 non-PED patients (Table 2, Fig 1B). Other than a statistically but probably not clinically significant difference in patient age (56.7±12.8 vs. 57.9±12.3 years, p = 0.04), the patient groups were well balanced in terms of sex (p = 0.9), payor type (p = 0.5), CCI group (p = 0.9), length of stay (p = 0.9), and rate of routine discharge disposition (p = 0.5). A reduced likelihood for repeat embolization was seen after PED relative to non-PED treatment at 90 days (2.7% vs. 7.9%, p<0.001), 180 days (4.7% vs. 10.3%, p<0.001), 1 year (7.1% vs. 13.1%, p<0.0001), and 2 years (8.1% vs. 14.7%, p<0.0001). The rate of readmission for repeat embolization was lower for PED compared with non-PED at all time points (p<0.05).

**Table 2:** Propensity score–matched analysis of patients with intracerebral aneurysms treated endovascularly.

Variable	PED N = 679	Non-PED N = 1918	P-value
Age (mean±STD; median), years	56.7 ± 12.8; 57	57.9 ±12.3; 58	0.04
Sex (% total), male	113 (16.6)	324 (16.9)	0.9
Payor (% total)			0.5
Commercial	351 (51.7)	936 (48.8)	
Medicaid	80 (11.8)	221 (11.5)	
Medicare	220 (32.4)	687(35.8)	
Other	28 (4.1)	75 (3.9)	
Charlson score group			0.9
0	457 (67.3)	1300 (67.8)	
1	129 (19.0)	353 (18.4)	
2+	93 (13.7)	265 (13.8)	
Length of stay (mean±STD; median), days	3.5 ± 3.7; 2	3.5 ± 4.3; 2	0.9
Routine discharge disposition	628 (92.5)	1752 (91.5)	0.5
All-cause readmissions including embolization			
30 days	50 (7.4)	150 (7.8)	0.8
90 days	81 (11.9)	316 (16.5)	0.005
180 days	113 (16.6)	403 (21.0)	0.02
1 year	151 (22.2)	503 (26.2)	0.05
2 years	170 (25.0)	579 (30.2)	0.01
Readmission for repeat embolization			
30 days	6 (0.9)	36 (1.9)	0.1
90 days	18 (2.7)	152 (7.9)	<0.001
180 days	32 (4.7)	198 (10.3)	<0.001
1 year	48 (7.1)	251 (13.1)	<0.001
2 years	55 (8.1)	282 (14.7)	<0.001

**Fig 2:**
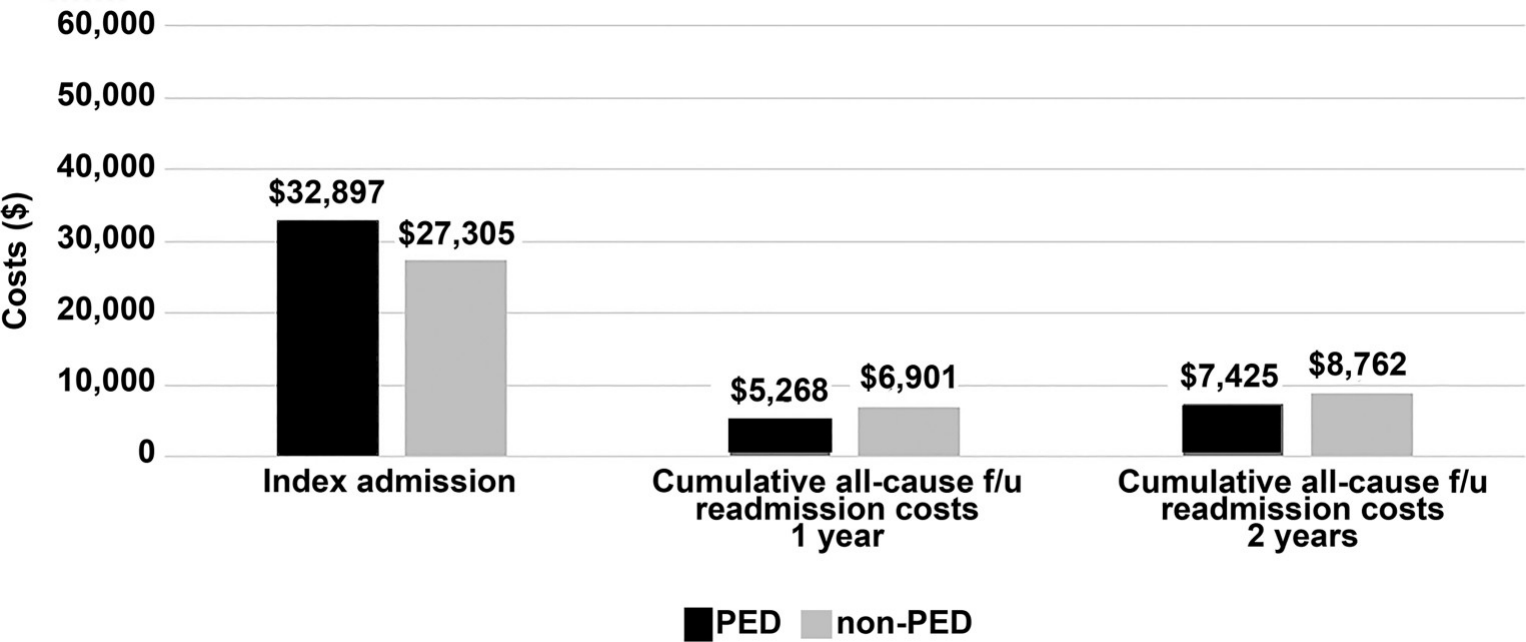
Mean internal hospital costs for the initial embolization admission and cumulative follow-up all-cause readmissions to the same hospital through one- and two-year follow-up in PED and non-PED patients. Costs include emergency department visits and readmission hospital costs including repeat embolization-related costs). PED = Pipeline Embolization Device.

The mean difference in initial hospitalization cost between PED and non-PED treatment modalities was significant when comparing the cost of admission for the initial index embolization and when evaluating cumulative readmission and emergency department visit costs over 1- and 2-year follow-up (Fig 2). Patients with PED treatment had higher initial hospitalization costs relative to non-PED ($32,897±17,384 vs. $27,305±15,942, p<0.001). Among the entire study cohort, cumulative follow-up emergency department visit and readmission costs were no different for patients with PED relative to non-PED treatment at 1 year ($5,268±15,440 vs. 6,901±18,887, p = 0.061) but were significantly lower at 2 years ($7425±28027 vs. 8762±22501, p = 0.021, Fig 2). This follow-up difference in cost directly reflects the lower incidence in all-cause readmissions among patients initially treated with PED. Separately, among patients with a readmission specifically for retreatment, the mean retreatment hospitalization cost was equivalent across cohorts at 2 years for PED vs. non-PED groups ($33,083±21,416 vs. 29,306±21,438, p = 0.096).

## Discussion

### Study findings

In this study, we found that patients who underwent PED treatment of unruptured intracranial aneurysms were less likely to require retreatment at 90 days after index aneurysm treatment, and this benefit lasted through 2 years of follow-up (range: PED: 2.7–8.1% vs. non-PED: 5.7–11.6%). These findings remained even after adjusting for patient risk factors via PS matching (range: PED: 2.7–8.1% vs. non-PED: 7.9–14.7%). Furthermore, there were fewer all-cause readmissions, which included emergency room visits or admission to the hospital because of aneurysm-related complaints or complications as well as other hospitalizations, among patients treated with PED. Finally, although initial treatment costs were higher for PED treatment ($32,897 vs. $27,305), cumulative follow-up readmission costs at 2 years (inclusive of patients with no readmission and/or no retreatment) were significantly lower for patients with initial PED relative to non-PED treatment, reflecting the lower incidence of readmissions for retreatment in the PED group.

The higher initial costs associated with PED also need to be viewed in the context of size and location of aneurysms, which cannot be captured through PHD data. Because the initial FDA approval for PED was for larger aneurysms, it is very likely that the PED group in our data had a much higher proportion of larger aneurysms relative to the non-PED group. PED has already been shown to be more cost effective for larger aneurysms even for procedure costs, with a 27.1% cost reduction per millimeter of aneurysm treated because of the higher cost of implants (stent(s), coils) with the increasing size of the aneurysm [20].

This study represents a real-world, multicenter comparison of PED utilization for the treatment of intracranial aneurysms with coil embolization. Follow-up costs and readmission rates were factored as potential metrics to better understand these two treatment modalities. This study also demonstrated significant cost heterogeneity (i.e., wide standard deviations) for the use of PED and non-PED technologies. This variability of inpatient costs likely depends on specific patient factors such as aneurysm size and location as well as intensive care unit length of stay, but it opens the promise of further improving resource utilization to drive down costs and standardize care.

### Baseline demographics

The demographics of patients in this study reflect groups treated by endovascular therapy. The mean patient age and sex distributions were similar to previous trials of coiling or PED [9, 11, 13, 15]. Interestingly, there were no differences in the initial hospitalization length of stay between groups. Prior studies have shown both shorter length of stays [18, 21] and similar-to-longer length of stays [22, 23] compared with this study. Given the heterogeneity of providers, patients, and hospitals included within the PHD, there was significant variability in length of stay, a factor widely known to affect healthcare costs [23]. The majority of patients were discharged home, similar to these other studies [9, 11, 13, 15].

### Readmissions and retreatment

Several studies have reported on the safety profile and efficacy of PED for the treatment of intracranial aneurysms. McDonald and colleagues used the PHD to analyze 279 patients with unruptured aneurysms who specifically underwent PED treatment from 2011 to 2013 [18]. Adverse outcomes included in-hospital mortality in 2 patients (0.7%), discharge to long-term care in 22 patients (7.9%), ischemia in 14 cases (5.0%), hemorrhagic complications in 4 cases (1.4%), and postoperative neurological complications in 9 cases (3.2%). In total, 13% of patients experienced some form of postoperative complication. Several meta-analyses of PEDs have demonstrated morbidity rates of 5–7% and mortality rates of 2.5–5% [24, 25]. Our current study adds to the prior literature by evaluating follow-up retreatment rates from PEDs, which were lower than for non-PED treatments.

The inclusion of follow-up in the calculus of treatment efficacy and cost has been recently described for PEDs. The PUFS (Pipeline for Uncoilable or Failed Aneurysms) trial showed complete occlusion at 1-year follow-up in 79 of 91 patients, with 6 of 107 patients having major stroke or death (the study’s primary safety endpoint) [15]. Follow-up of the PUFS trial at 3 years showed aneurysm occlusion in 71 of 76 patients with no additional occurrences of the safety endpoints [15]. The efficacy of PEDs has been supported by multiple other groups including a number of study registries [18, 21, 22, 26–28]. The results of our study support a lower retreatment rate after PED embolizations as compared with non-PED treatments.

### Hospital cost

Previous studies have examined the cost of PED and coiling treatments [20, 23, 27, 29]. Colby et al. [20] investigated the costs of coiling versus flow diversion treatment of anterior circulation aneurysms and noted that total costs were lower among patients treated with flow diversion compared with those treated with stent coiling. Evaluation of 30 PED and 30 non-PED patients showed a lower cost of implants ($13,175±726 vs. $19,069±2,015, p = 0.013) and a lower total procedure cost ($16,445±735 vs. $22,145±2,022, p = 0.02) in the PED group. Another cost-focused study used results from the University of Utah Value Driven Outcome tool to analyze patient costs, evaluate cost drivers, and find ways to reduce cost expenditures in aneurysm treatment [23]. Including both ruptured and unruptured aneurysms, 514 aneurysm treatments (273 clipped, 102 coiled, 139 PED) were analyzed by aneurysm size, location, and patient factors. Coiling and PED cases cost on average 1.5× and 1.2× more than clipping, respectively. A multivariate analysis showed that rupture status, procedure type, ASA status, discharge disposition, and year of surgery all were independent predictors of cost [23]. By limiting our data set to patients with unruptured aneurysms in this present study, we were able to reduce the confounding influence introduced by rupture status given the increased costs associated with management of patients with subarachnoid hemorrhage. Moreover, our data set encompasses multiple centers and providers, thus increasing its generalizability. Others have reiterated this finding by showing that PEDs may potentially be less costly than coiling, especially when considering retreatment [27].

### Strengths and limitations

The strengths of this study are that the PHD is a large, well-documented database that, by design, comprises a random sample of the population presenting to both community and teaching/academic hospitals in disparate regions of the country, making the findings more generalizable to the population as a whole than prior single-center cohort studies. The main limitation of this analysis is the cross-sectional nature of the PHD data set, which only allows for follow-up evaluation of patients returning to the same hospital where their initial treatment was performed. Given this limitation, our study may underestimate retreatment rates among both cohorts of patients. Recurrence after treatment is also more likely in larger aneurysms compared with smaller aneurysms [30]. Given the likelihood that PED was performed for larger aneurysms than in the non-PED group, PED is likely even more cost-effective when compared for similar sized aneurysms. The reduced risk of recanalization and corresponding reduced follow-up imaging may result in further reduction of costs with PED, which is not factored in the current analysis. In addition, the PHD does not allow for distinguishing between retreatment of the index aneurysm versus a separate intracranial aneurysm, and thus, we cannot be certain that we are analyzing the true rate of retreatment of the index aneurysm in this data set. Finally, there were no data pertaining to the specifics of the treated index aneurysm in terms of size or morphology, which potentially represents a confounding factor in predicting successful occlusion and cost [23]. The indications for PED were recently expanded to include small and medium wide-necked (neck width ≥4 mm or dome-to-neck ratio <2) saccular or fusiform intracranial aneurysm arising from a parent vessel with a diameter ≥2.0 mm and ≤5.0 mm. Further recent refinements in PED technology (i.e., Pipeline Flex and Pipeline Shield) may be correlated with additional reductions in procedure-related morbidity and mortality with a significantly greater ease of deployment, which would not have been captured in earlier years of our present study.[31]Additional clinical factors pertaining to the actual embolization procedure and follow-up were not evaluated, given the lack of imaging and other clinical details not included in this administrative data set. Thus, there was no standardization in terms of treatment procedures, post-treatment management, follow-up, readmission protocols, or occlusion thresholds for aneurysm retreatment. Nonetheless, the PHD offers the best ability to evaluate readmission and retreatment among available administrative databases based on the breakdown by CPT and ICD codes for individual patients. It remains unlikely that a different database would result in more granular results; instead, additional cohort and institutionalized studies may be required to better understand the findings of this study.

## Conclusions

For unruptured aneurysms in a multicenter evaluation, these study results suggest a lower retreatment rate for patients after PED treatment compared with non-PED treatments. When controlling for confounding variables, there were also lower rates of readmission and emergency department reevaluation at 90 days, 180 days, 1 year, and 2 years after index aneurysm treatment. Despite higher initial costs for PEDs, cumulative follow-up emergency department and inpatient costs, inclusive of all patients regardless of readmission or retreatment status, were significantly lower at two years amongst patients treated with PEDs. The findings of our study using the multicenter PHD suggest not only an important role for PEDs in the modern armamentarium for intracranial aneurysms but one that may prove to be associated with less burden in terms of utilization of valuable healthcare and economic resources.

## References

[1] Chalouhi N, Hoh BL, Hasan D. Review of cerebral aneurysm formation, growth, and rupture. Stroke. 2013;44(12):3613–22. 10.1161/STROKEAHA.113.002390 24130141

[2] Li MH, Chen SW, Li YD, Chen YC, Cheng YS, Hu DJ, et al Prevalence of unruptured cerebral aneurysms in Chinese adults aged 35 to 75 years: a cross-sectional study. Ann Intern Med. 2013;159(8):514–21. 10.7326/0003-4819-159-8-201310150-00004 24126645

[3] Chen ML, Gupta A, Chatterjee A, Khazanova D, Dou E, Patel H, et al Association between unruptured intracranial aneurysms and downstream stroke. Stroke. 2018;49(9):2029–33. 10.1161/STROKEAHA.118.021985 30354970 PMC6205209

[4] Andaluz N, Zuccarello M. Recent trends in the treatment of cerebral aneurysms: analysis of a nationwide inpatient database. J Neurosurg. 2008;108(6):1163–9. 10.3171/JNS/2008/108/6/1163 18518722

[5] Naggara ON, White PM, Guilbert F, Roy D, Weill A, Raymond J. Endovascular treatment of intracranial unruptured aneurysms: systematic review and meta-analysis of the literature on safety and efficacy. Radiology. 2010;256(3):887–97. 10.1148/radiol.10091982 20634431

[6] Zhao J, Lin H, Summers R, Yang M, Cousins BG, Tsui J. Current treatment strategies for intracranial aneurysms: an overview. Angiology. 2018;69(1):17–30. 10.1177/0003319717700503 28355880 PMC5724574

[7] Currie S, Mankad K, Goddard A. Endovascular treatment of intracranial aneurysms: review of current practice. Postgrad Med J. 2011;87(1023):41–50. 10.1136/pgmj.2010.105387 20937736

[8] Pierot L, Spelle L, Leclerc X, Cognard C, Bonafe A, Moret J. Endovascular treatment of unruptured intracranial aneurysms: comparison of safety of remodeling technique and standard treatment with coils. Radiology. 2009;251(3):846–55. 10.1148/radiol.2513081056 19318586

[9] Molyneux A, Kerr R, Stratton I, Sandercock P, Clarke M, Shrimpton J, et al International Subarachnoid Aneurysm Trial (ISAT) of neurosurgical clipping versus endovascular coiling in 2143 patients with ruptured intracranial aneurysms: a randomised trial. Lancet. 2002;360(9342):1267–74. 10.1016/s0140-6736(02)11314-6 12414200

[10] Pierot L, Cognard C, Anxionnat R, Ricolfi F, Investigators C. Ruptured intracranial aneurysms: factors affecting the rate and outcome of endovascular treatment complications in a series of 782 patients (CLARITY study). Radiology. 2010;256(3):916–23. 10.1148/radiol.10092209 20720074

[11] McDougall CG, Spetzler RF, Zabramski JM, Partovi S, Hills NK, Nakaji P, et al The Barrow Ruptured Aneurysm Trial. J Neurosurg. 2012;116(1):135–44. 10.3171/2011.8.JNS101767 22054213

[12] Rajah G, Narayanan S, Rangel-Castilla L. Update on flow diverters for the endovascular management of cerebral aneurysms. Neurosurg Focus. 2017;42(6):E2 10.3171/2017.3.FOCUS16427 28565980

[13] Nelson PK, Lylyk P, Szikora I, Wetzel SG, Wanke I, Fiorella D. The pipeline embolization device for the intracranial treatment of aneurysms trial. AJNR Am J Neuroradiol. 2011;32(1):34–40. 10.3174/ajnr.A2421 21148256 PMC7964968

[14] Becske T, Potts MB, Shapiro M, Kallmes DF, Brinjikji W, Saatci I, et al Pipeline for uncoilable or failed aneurysms: 3-year follow-up results. J Neurosurg. 2017;127(1):81–8. 10.3171/2015.6.JNS15311 27739944

[15] Becske T, Kallmes DF, Saatci I, McDougall CG, Szikora I, Lanzino G, et al Pipeline for uncoilable or failed aneurysms: results from a multicenter clinical trial. Radiology. 2013;267(3):858–68. 10.1148/radiol.13120099 23418004

[16] Orru E, Roccatagliata L, Cester G, Causin F, Castellan L. Complications of endovascular treatment of cerebral aneurysms. Eur J Radiol. 2013;82(10):1653–8. 10.1016/j.ejrad.2012.12.011 23332977

[17] Campi A, Ramzi N, Molyneux AJ, Summers PE, Kerr RS, Sneade M, et al Retreatment of ruptured cerebral aneurysms in patients randomized by coiling or clipping in the International Subarachnoid Aneurysm Trial (ISAT). Stroke. 2007;38(5):1538–44. 10.1161/STROKEAHA.106.466987 17395870

[18] McDonald RJ, McDonald JS, Kallmes DF, Lanzino G, Cloft HJ. Periprocedural safety of Pipeline therapy for unruptured cerebral aneurysms: Analysis of 279 Patients in a multihospital database. Interv Neuroradiol. 2015;21(1):6–10. 10.1177/1591019915576289 25934768 PMC4757207

[19] Premier Applied Sciences. Premier Healthcare Database White Paper: Data That Informs and Performs. 2018 July 28. [Cited 2019 September 1]. Available from: https://products.premierinc.com/downloads/PremierHealthcareDatabaseWhitepaper.pdf.

[20] Colby GP, Lin LM, Paul AR, Huang J, Tamargo RJ, Coon AL. Cost comparison of endovascular treatment of anterior circulation aneurysms with the pipeline embolization device and stent-assisted coiling. Neurosurgery. 2012;71(5):944–48; discussion 948–50. 10.1227/NEU.0b013e3182690b8b 22806083

[21] Lin LM, Colby GP, Kim JE, Huang J, Tamargo RJ, Coon AL. Immediate and follow-up results for 44 consecutive cases of small (<10 mm) internal carotid artery aneurysms treated with the pipeline embolization device. Surg Neurol Int. 2013;4:114 10.4103/2152-7806.117711 24083050 PMC3779399

[22] Yu SC, Kwok CK, Cheng PW, Chan KY, Lau SS, Lui WM, et al Intracranial aneurysms: midterm outcome of pipeline embolization device—a prospective study in 143 patients with 178 aneurysms. Radiology. 2012;265(3):893–901. 10.1148/radiol.12120422 22996749

[23] Twitchell S, Abou-Al-Shaar H, Reese J, Karsy M, Eli IM, Guan J, et al Analysis of cerebrovascular aneurysm treatment cost: retrospective cohort comparison of clipping, coiling, and flow diversion. Neurosurg Focus. 2018;44(5):E3 10.3171/2018.1.FOCUS17775 29712525

[24] Brinjikji W, Murad MH, Lanzino G, Cloft HJ, Kallmes DF. Endovascular treatment of intracranial aneurysms with flow diverters: a meta-analysis. Stroke. 2013;44(2):442–7. 10.1161/STROKEAHA.112.678151 23321438

[25] Arrese I, Sarabia R, Pintado R, Delgado-Rodriguez M. Flow-diverter devices for intracranial aneurysms: systematic review and meta-analysis. Neurosurgery. 2013;73(2):193–9; discussion 9–200. 10.1227/01.neu.0000430297.17961.f1 23624409

[26] Lopes DK, Jang DK, Cekirge S, Fiorella D, Hanel RA, Kallmes DF, et al Morbidity and mortality in patients with posterior circulation aneurysms treated with the Pipeline Embolization Device: A subgroup analysis of the International Retrospective Study of the Pipeline Embolization Device. Neurosurgery. 2018;83(3):488–500. 10.1093/neuros/nyx467 28945879

[27] Malhotra A, Wu X, Miller T, Matouk CC, Sanelli P, Gandhi D. Comparative effectiveness analysis of Pipeline device versus coiling in unruptured aneurysms smaller than 10 mm. J Neurosurg. 2019:1–9. 10.3171/2018.8.JNS181080 30641830

[28] Phillips TJ, Wenderoth JD, Phatouros CC, Rice H, Singh TP, Devilliers L, et al Safety of the pipeline embolization device in treatment of posterior circulation aneurysms. AJNR Am J Neuroradiol. 2012;33(7):1225–31. 10.3174/ajnr.A3166 22678845 PMC7965498

[29] Gonda DD, Khalessi AA, McCutcheon BA, Marcus LP, Noorbakhsh A, Chen CC, et al Long-term follow-up of unruptured intracranial aneurysms repaired in California. J Neurosurg. 2014;120(6):1349–57. 10.3171/2014.3.JNS131159 24724850

[30] Petr O, Brinjikji W, Cloft H, Kallmes DF, Lanzino G. Current trends and results of endovascular treatment of unruptured intracranial aneurysms at a single institution in the flow-diverter era. AJNR Am J Neuroradiol. 2016;37(6):1106–13. 10.3174/ajnr.A4699 26797138 PMC7963541

[31] Le EJ, Miller T, Serulle Y, Shivashankar R, Jindal G, Gandhi D. Use of Pipeline Flex is associated with reduced fluoroscopy time, procedure time, and technical failure compared with the first-generation Pipeline embolization device. J Neurointerv Surg. 2017;9(2):188–91. 10.1136/neurintsurg-2016-012261 26962044

